# Influence of Movement Restriction During the COVID-19 Pandemic on Uptake of DMPA-SC and Other Injectable Contraceptive Methods in Nigeria

**DOI:** 10.2147/OAJC.S494588

**Published:** 2025-05-26

**Authors:** Adewole Adebola Adefalu, Olufunke Abimbola Bankole, Funmilayo Olabode, Mojuba Bimbo Afolabi, Miranda Atare Buba, Victor Dafe, Mishael Nnanna Kalu, Emily Watkins

**Affiliations:** 1 JSI Research & Training Institute, Inc., Atlanta, GA, USA; 2 JSI Research & Training Institute, Inc., Abuja, FCT, Nigeria; 3 JSI Research & Training Institute, Inc., Washington, DC, USA

**Keywords:** COVID-19, family planning, contraception, depot medroxyprogesterone acetate, injectable contraceptive methods

## Abstract

**Introduction:**

The COVID-19 pandemic caused significant disruptions to sexual and reproductive health (SRH) services globally, with a pronounced impact on low- and middle-income countries like Nigeria. This study investigates how COVID-19 travel restrictions influenced the uptake of depot medroxyprogesterone acetate subcutaneous (DMPA-SC) and other injectable contraceptives in Nigeria.

**Methods:**

This study analyzed 26 months of secondary logistics data from the national electronic Health Logistics Management Information System (e-HLMIS), covering 36 states and encompassing periods before, during, and after the implementation of travel restrictions. Statistical analyses, including one-way ANOVA and independent samples *t*-tests, were applied to assess trends in the consumption of DMPA-SC and compare them with other injectable contraceptives, such as intramuscular DMPA (DMPA-IM) and norethisterone enanthate (NET-EN).

**Results:**

Findings showed a significant increase in DMPA-SC consumption during the travel restriction period, with mean consumption rising from 57,187 units pre-restriction to 103,249 units during the restriction. This increase persisted post-restriction, with mean consumption reaching 124,561 units. While the use of other injectable contraceptives also rose during the pandemic, their growth did not sustain as consistently as DMPA-SC.

**Discussion:**

The results suggest that promoting self-administration of DMPA-SC was essential in maintaining contraceptive access when conventional healthcare services were disrupted. This study highlights the importance of adaptable healthcare delivery models, such as self-administration, in ensuring SRH service continuity during global crises. Additionally, it underscores the need for resilient supply chain management to secure contraceptive availability in emergencies, providing critical insights for policymakers and healthcare providers aiming to enhance SRH service resilience in future public health challenges.

## Plain language summary

The COVID-19 pandemic disrupted healthcare services globally, including in Nigeria, making it challenging for women to access family planning services. Many women in Nigeria rely on injectable contraceptives to prevent pregnancy, but pandemic-related travel restrictions and fears of the virus made it difficult to visit health clinics for these services.

This study investigated how the pandemic influenced the use of injectable contraceptives, particularly DMPA-SC, a type of injectable contraceptive that women can self-administer at home. Researchers analyzed data from health facilities across Nigeria, covering 26 months that included periods before, during, and after COVID-19 travel restrictions. They compared the use of DMPA-SC with other injectables, like DMPA-IM and norethisterone enanthate (NET-EN), which require administration by a healthcare provider.

The study found that more women began using DMPA-SC during the travel restrictions, likely due to its self-administration feature, which provided autonomy and convenience when clinic access was limited. Even after the restrictions were lifted, the use of DMPA-SC continued to grow, suggesting that many women preferred this method once they experienced its benefits. While the use of other injectables also increased during the pandemic, the rise in DMPA-SC use was more significant and sustained.

These findings highlight the importance of offering self-administered contraceptives to ensure women can maintain control over their reproductive health during crises. They also emphasize the need for strong supply chains to make contraceptives consistently available. Supporting self-administered options like DMPA-SC empowers women and ensures continuous access to family planning, even during emergencies.

## Introduction

Global and regional health emergencies can severely disrupt sexual and reproductive health services, including family planning, especially in low- and middle-income countries like Nigeria. The COVID-19 pandemic, in particular, has significantly reduced the use of these services globally, with a 10% proportional decline in 132 low- and middle-income countries.^1^ Factors contributing to this decline include supply chain disruptions, fear of virus transmission at health facilities, and reallocating healthcare resources to address the pandemic.^2^ In Nigeria, pre-existing vulnerabilities within the healthcare system were exacerbated by the pandemic, leading to a particularly dire situation.^3^

Pandemics worsen existing barriers to healthcare access, such as quarantines, staff shortages, and fear of disease, threatening progress in family planning.^4^ Historical pandemics like the Ebola outbreak in West Africa show how reproductive health services are impacted during health crises. Between 2014 and 2016, teenage pregnancies in Sierra Leone surged by 24%, and the uptake of contraceptives and maternal health services dropped significantly.^5^,^6^ Bietsch et al^7^ also documented declines in family planning services in Sierra Leone and Liberia during Ebola’s first six months, underscoring the need for resilient healthcare systems.

Pre-existing disparities in healthcare access, rooted in socioeconomic inequalities, gender norms, and geographical challenges, also worsened during the COVID-19 pandemic, disproportionately affecting marginalized groups.^8^ The pandemic highlighted how gender, income, and geography intersect to create complex barriers to reproductive health services.^9^ Further complicating these issues, the incidence of sexual violence often increases during disasters, resulting in more unwanted pregnancies and sexually transmitted infections.^10^,^11^ The United Nations Population Fund (UNFPA) described the surge in gender-based violence during the COVID-19 pandemic as a “shadow pandemic”, which amplified unmet family planning needs.^12^

Access to family planning allows individuals to make informed reproductive health decisions during crises. However, the uptake of specific methods varies under such conditions. For example, Bietsch et al^7^ found that implants and injectables faced reduced uptake during COVID-19, whereas oral contraceptive pills (OCPs) were more popular due to the autonomy they afford.^13^,^14^

Women using injectable contraceptives experience heightened barriers during pandemics due to disrupted supply chains, leading to stockouts at health facilities.^15^ The COVID-19 pandemic’s rapid onset posed new challenges to supply chains, and response measures like movement restrictions and travel bans, though essential, inadvertently impacted essential services such as family planning.^16^

Nigeria’s family planning supply chain, reliant on central procurement and funded every four months, already faced logistical and procurement bottlenecks, causing frequent stockouts.^17^ The pandemic further threatened this delivery system, prompting the World Health Organization (WHO) to emphasize the importance of robust supply chain systems that can continue to provide essential health products during emergencies.^18^

Despite gains in family planning, Nigeria has struggled to meet the growing demand, particularly in rural and underserved areas. From 2013 to 2018, demand among women of reproductive age rose from 29% to 38%, yet unmet needs increased from 13% to 20%.^19^ The limited availability of modern contraceptives worsens this gap.^20^

Nigeria has taken steps to increase family planning access, such as introducing depot medroxyprogesterone acetate subcutaneous (DMPA-SC), a contraceptive suitable for self-administration and delivery by lower-cadre staff. This intervention proved valuable during the pandemic, as DMPA-SC’s self-injection option facilitated contraceptive access amidst limited healthcare availability.^21–23^ Many experts believe DMPA-SC could be a “game-changer” in low-resource settings due to its potential for self-injection and ease of use.^21^,^24^ Following Nigeria’s 2012 Family Planning Summit commitment, a strategic plan guided DMPA-SC’s introduction with government and international donor support.^25^

The efforts yielded gains before COVID-19, making DMPA-SC accessible across most public sector channels.^26^ To ensure continuous supplies of contraceptive methods, the Ministry of Health provided official passes for vehicles to distribute all FP commodities from the central contraceptive warehouse to the states and for health facilities to continue offering services to women and their families during the period of movement restriction. This way, Nigeria could avoid a complete stockout of these commodities and continue to provide services to women.

Emerging studies report a notable decline in family planning services during COVID-19, particularly for injectable contraceptives, due to movement restrictions and fear of infection.^16^,^27^ These findings emphasize the importance of strengthening supply chains and exploring alternative delivery methods, such as self-injection, to ensure contraceptive service continuity during health crises. However, there is limited research on how the pandemic affected contraceptive method choice, particularly for injectable options.^28^ No studies have also specifically examined the direct impact of COVID-19 movement restrictions on injectable contraceptives or DMPA-SC, indicating a significant research gap.^27^

This study aims to 1) analyze DMPA-SC and other injectable contraceptive trends before, during, and after COVID-19 travel restrictions; 2) assess COVID-19 travel restrictions’ effect on DMPA-SC use; 3) examine if significant differences exist in injectable contraceptive use across the restriction periods; and 4) explore how travel restrictions relate to supply chain factors. Insights from this study can inform policy and service delivery strategies to enhance DMPA-SC access in Nigeria and offer guidance to other countries facing similar challenges or aiming to introduce new family planning products during emergencies.

## Materials and Methods

This study used secondary data extracted from the Access Collaborative DMPA-SC dashboard. The dashboard draws data from Nigeria’s national electronic Health Logistics Management Information System (e-HLMIS), an electronic platform designed to provide detailed visibility into health logistics and stock levels at health facilities nationwide.^29^ The e-HLMIS allows for near-real-time data collection, collation, storage, and analysis, supporting inventory management and decision-making at federal, state, and local government levels.

The dataset encompassed 26 months of logistics management information from 36 states, covering 12 months before, six months during, and eight months after the enforcement of travel restrictions in Nigeria. Consumption data was used as a proxy for the uptake of contraceptive methods because it reflects the quantities of contraceptives distributed or utilized within a specific period and geographic location. The consumption data is often routinely collected through supply chain management systems, making it a readily available dataset for inferring trends in contraceptive use. This data can be disaggregated by method type, location, and timeframe to estimate changes in uptake patterns.

The inclusion criteria for data used in this study were: 1) facilities reporting data on injectable contraceptives (eg, DMPA-SC, DMPA-IM, NET-EN) during the pre-restriction, restriction, and post-restriction periods; 2) data collected within a specified and continuous time frame (eg, 26 months) that covers pre-pandemic, restriction, and post-pandemic periods to enable trend analysis; 3) all reported injectable contraceptives, including DMPA-SC, DMPA-IM, and NET-EN; 4) data from facilities across the entire study area (eg, 36 states of Nigeria) to ensure comprehensive regional representation; and 5) data on the number of units of contraceptives dispensed to clients (not stock at facilities) to accurately reflect actual uptake. The authors have excluded data on other contraceptive methods (eg, implants, oral contraceptives, condoms) and those from periods outside the defined pre-restriction, restriction, and post-restriction windows.

Data extracted included details on commodity stock levels, receipts, consumption, and losses. The analysis utilized SPSS 27.0, with results presented in frequency tables, charts, and inferential statistics to identify trends and patterns in contraceptive use.

In preparing this manuscript, the AI tool OpenAI’s ChatGPT (version GPT-4) was used to generate text and refine sections of the methodology, discussion, and overall structure of the research proposal. The tool was employed to streamline the writing process, ensuring clarity and coherence in presenting complex ideas. All content generated through the AI tool was thoroughly reviewed and edited by the authors to ensure accuracy, originality, and compliance with the research objectives. No data analysis or decision-making related to research findings was conducted using the AI tool.

A comparative analysis was performed to evaluate differences in DMPA-SC utilization compared to two other injectable contraceptives: intramuscular depot medroxyprogesterone acetate (DMPA-IM) and norethisterone enanthate (NET-EN). This comparison aimed to assess variations in utilization patterns and the impact of the pandemic on different injectable methods. The analysis also integrated demographic indicators and logistical data, such as the availability of commodities, stock received by service delivery points (SDPs), and consumption rates, to provide a comprehensive overview of how self-administration and policy changes influenced contraceptive access during the pandemic.

Nigeria’s National Health Research Ethics Committee (NHREC) exempts the study from ethical approval. Section B (part C) on pages 4 and 5 of the “National Code of Health Research Ethics” legislation document details all exemptions. The board exempts all research that utilizes existing data, documents, records, pathological specimens, or diagnostic specimens if these sources are publicly available.

## Results

A two-period moving average trendline was utilized to simulate the anticipated consumption trend of DMPA-SC over a specified period. The analysis revealed that actual consumption mirrored expected consumption during the pre-COVID-19 travel restriction period. However, during the travel restriction period, actual consumption consistently exceeded the expected consumption, except for the April 2020 reporting period. A similar trend was observed for two other injectable products, characterized by sharp increases in consumption during the travel restriction period, followed by a slight decline in April 2020.

The mean consumption of DMPA-SC before, during, and after the travel restriction periods were 57,187 ± 14,959.76, 103,249 ± 14,498.33, and 124,561 ± 19,394, respectively (Table 1). A one-way ANOVA indicated a statistically significant difference in DMPA-SC consumption across the different periods (F(2,10) = 20.75, p < 0.0001). Post-hoc analysis (Table 2) using the Tukey’s test showed that consumption during the travel restriction period was significantly higher (103,249 ± 14,498.33, p = 0.01) compared to the pre-restriction period (57,187 ± 14,959.76) though considerably lower when compared to the post-restriction period (124,561 ± 19,394, p < 0.0001). Notably, no statistically significant difference existed between the consumption during the travel restriction and post-restriction periods.

**Table 1:** Analysis of Variance of DMPA-SC Consumption Across Time Periods (N=13)

Period	Mean	SD	F (df)	p-value
Pre-travel restriction	57,187.00	14,959.755	20 (2,10)	<0.0001
Travel restriction	103,249.00	14,498.332		
Post-travel restriction	124,561.00	19,394.463

**Table 2:** Post-Hoc Test Comparing DMPA-SC Mean Consumption Across Time Periods

Periods	Periods	Mean Difference	Std. Error	p-value	95% Confidence Interval
Pre	Intra	−46,062.000*	12,264.118	0.010	−79,681.57 to −12,442.43
Post	−67,374.000*	10,621.038	0.000	−96,489.40 to −38,258.60	
Intra	Pre	46,062.000*	12,264.118	0.010	12,442.43 to 79,681.57
Post	−21,312.000	12,264.118	0.239	−54,931.57 to 12,307.57	
Post	Pre	67,374.000*	10,621.038	0.000	38,258.60 to 96,489.40
Intra	21,312.000	12,264.118	0.239	−12,307.57 to 54,931.57	

**Note**: *p-values < 0.05.

**Figure 1:**
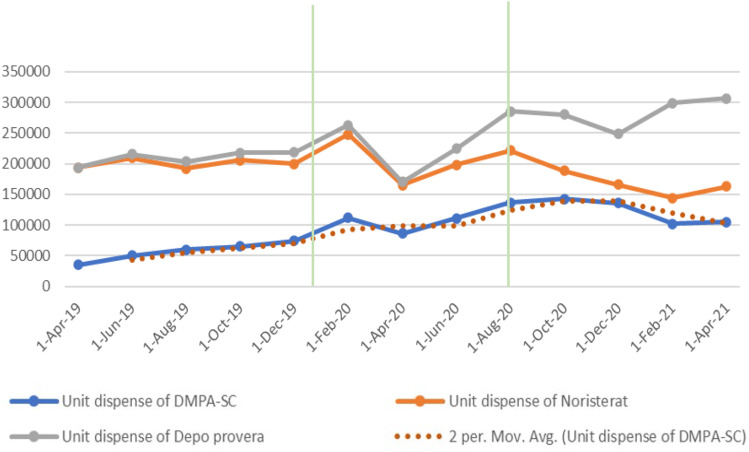
Comparison between the uptake of all injectable methods pre-, during, and post-restriction in travels.

Figure 1 illustrates the comparison of DMPA-SC consumption across pre-, intra-, and post-COVID-19 travel restriction periods. Univariate analysis indicated a steady rise in DMPA-SC consumption in the pre-COVID period, peaking at 41% growth between April 2019 and June 2019. A sharp increase in consumption, with a 50.2% period-on-period growth, was observed at the onset of COVID-19 travel restrictions in February 2020. This rise in consumption was followed by a 22% decline in April 2020 and subsequent increases of 28.3% and 23.2% during the travel restriction period in the following reporting periods. Post-restriction, consumption showed a slight 4% increase, followed by 4.4% and 25.2% declines in the subsequent reporting periods.

**Figure 2:**
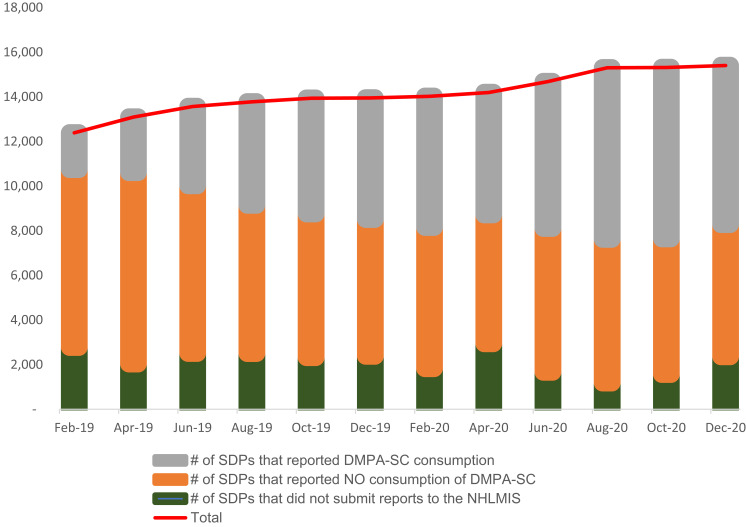
Systemic health facility reporting of DMPA-SC to the national HLMIS pre-, during, and post-restriction in travels.

Figure 2 depicts the fluctuations in the percentage of health facilities reporting DMPA-SC consumption. Despite the fluctuations, an increase in the absolute number of facilities reporting to the HLMIS was noted across pre-, during, and post-COVID-19 travel restriction periods. The percentage of facilities reporting DMPA-SC remained nearly constant relative to the pre-pandemic period, with minor dips in two bimonthly reporting cycles and a notable dip post-travel restriction.

An independent samples *t*-test was conducted to compare the mean consumption during periods of unrestricted movement versus travel restrictions. The consumption of DMPA-SC was higher during the travel restriction (103,249 ± 14,498 units) compared to periods of unrestricted movement (90,874 ± 39,083 units). Similarly, NET-EN injection consumption was higher during this period (204,006 ± 41,544 units). Conversely, DMPA-IM consumption was lower (219,335 ± 46,477 units) than during unrestricted movement. The differences in means were statistically significant for all products: t(9.8) = 0.829, p = 0.427 (DMPA-SC), p = 0.353 (DMPA-IM), and p = 0.416 (NET-EN) (Table 3).

**Table 3:** Independent Samples *T*-Test for Mean Consumption of Injectables Between Travel Restriction and Unrestricted Movement Periods

Measure	Mean	t-value	p-value	95% Confidence Interval
DMPA-SC Consumption		0.829	0.427	−39,623.49 to 64,373.49
COVID Travel Restriction	103,249 ± 14,498			
Open Movement	90,874 ± 39,083			
DMPA-IM Consumption		−0.969	0.353	−90,100.06 to 35,004.86
COVID Travel Restriction	219,335 ± 46,477			
Open Movement	246,882 ± 42,404			
NET-EN Consumption		0.845	0.416	−24,942.05 to 56,042.65
COVID Travel Restriction	204,006 ± 41,544			
Open Movement	188,455 ± 23,897			

A multivariate linear regression analysis demonstrated a statistically significant relationship between the percentage of stock-outs and the couple-year protection (CYP) for DMPA-SC (p = 0.05). The model accounted for a substantial portion of the variance in CYP (R²_adjusted = 0.910, F(4,13) = 20.23, p = 0.01) (Table 4). The regression coefficient (B = 330.98, 95% CI [4.15, 657.8]) indicated that for each percentage-point increase in stock-outs, there was an average increase of 330 CYP units.

**Table 4:** Multivariate Linear Regression Results for Factors Influencing Couple-Year Protection

Variable	Coefficient (B)	95% Confidence Interval (CI)	p-value
Percentage of Stock-Outs	330.98	[4.15, 657.80]	0.05
Stock on Hand (Units)	0.14	[0.05, 0.23]	<0.01
Quantity Received (Units)	0.15	[0.08, 0.22]	<0.01
Travel Restrictions	−15.23	[−50.78, 20.32]	0.37

**Notes**: Adjusted R²: 0.910, F-statistic: 20.23 (p = 0.01).

Additionally, there was an increase in CYP, corresponding to the rise in stock on hand of 0.14 and units of stock received in SDPs of 0.15 points. These findings highlight the interconnectedness of stock availability and contraceptive protection coverage, suggesting that facilities with higher stock levels and timely resupply were better positioned to sustain contraceptive uptake and meet user needs during the study period.

However, the analysis showed that the travel restrictions did not explain a significant variance in CYP (p = 0.37), suggesting that the primary drivers of CYP variability were internal supply chain factors rather than external contextual variables. These findings highlight the influence of supply chain performance—specifically stock availability—on contraceptive coverage, with stock-outs as a critical determinant of CYP trends during the study period.

Variables were selected based on their theoretical and practical significance in influencing CYP, with a focus on supply chain dynamics and external contextual factors. These variables represent key determinants of contraceptive availability and access at the service delivery points (SDPs). Only variables for which reliable and complete data were available from the national electronic Health Logistics Management Information System (e-HLMIS) were considered for inclusion. This ensured the robustness and accuracy of the analysis. Similarly, travel restrictions were included as an external contextual variable due to their significant impact on healthcare logistics during the COVID-19 pandemic. Variables demonstrating at least marginal significance in univariate analyses were considered for inclusion in the multivariable model to ensure they contributed meaningfully to explaining the variance in CYP.

The final multivariate model included these four variables after meeting the criteria above. The adjusted R² value of 0.910 indicates that the model explained 91% of the variance in CYP, with the F-statistic (F[4,13] = 20.23, p = 0.01) confirming the overall significance of the model.

## Discussion

The COVID-19 pandemic significantly impacted the consumption trends of DMPA-SC and other injectable contraceptive methods due to changes in healthcare access and the promotion of self-administration. Recent studies indicate a notable increase in DMPA-SC consumption during the COVID-19 pandemic. This trend was primarily driven by the promotion of self-administration to ensure continuity of contraceptive use despite restricted access to healthcare facilities. Research highlights that self-injected DMPA-SC became a crucial method for maintaining contraceptive use during the pandemic, with several countries integrating self-injection into their national family planning programs.^30–32^ In Nigeria, policy support was crucial for the scale-up of DMPA-SC. A study on the policy environment in Nigeria highlighted the role of enabling policies in facilitating the rapid uptake of DMPA-SC during the pandemic, ensuring that women could access contraceptives despite movement restrictions.^33^ Similar trends were observed globally, with countries like the Democratic Republic of the Congo (DRC) successfully using community-based distribution to ensure women in remote areas could access DMPA-SC.^34^

Furthermore, studies from Kenya and Malawi showed that self-administration increased contraceptive continuation rates compared to provider-administered methods.^35–37^ The significant increase in DMPA-SC consumption during travel restrictions indicates a shift in contraceptive use patterns driven by the necessity for self-administered options. Studies by PATH and UNFPA illustrate that self-injection of DMPA-SC became vital in family planning programs in various countries, such as Kenya and Nigeria, where large-scale distributions were implemented to meet the rising demand.^30^,^32^

Similarly, WHO’s research on self-injection pilot projects in multiple regions confirmed the positive impact of self-administration on maintaining contraceptive use during restricted access periods.^37^ These trends highlight the adaptability of contraceptive practices in response to public health emergencies and underscore the importance of flexible healthcare delivery models. Additional studies have shown that task-shifting policies and community-based interventions were critical in these efforts, demonstrating the effectiveness of these strategies in various contexts.^33^,^34^ The observed trends in DMPA-SC consumption during the COVID-19 pandemic emphasize the importance of self-administered contraceptives in ensuring continuity of care during healthcare disruptions. By adopting flexible healthcare delivery models and promoting self-care interventions, policymakers can enhance the resilience of contraceptive services, providing sustained access for women worldwide.

Movement restrictions during the COVID-19 pandemic influenced the availability and distribution of contraceptive supplies, affecting DMPA-SC consumption. Quantitative analyses confirmed significant variations in DMPA-SC consumption across different periods, with higher consumption during travel restrictions compared to the pre-restriction period and an even greater increase observed in the post-restriction period. Studies attributed this increase to the necessity for self-administered options when traditional healthcare services are disrupted.^30^,^37^ The promotion of self-administration was pivotal during the pandemic. The WHO emphasized the importance of self-care interventions, including self-injected contraceptives, to enhance women’s autonomy and ensure continued access to contraception.^30^,^37^

Data from health facilities showed fluctuations in DMPA-SC reporting but an overall increase in absolute numbers during the pandemic. These changes suggest disruptions in data reporting mechanisms and a focus on maintaining contraceptive supplies despite disruptions. However, challenges related to stockouts and supply chain disruptions persisted, highlighting the need for robust supply chains and effective stock management.^30^,^32^ Further studies indicate that countries with strong policy support and practical implementation strategies, such as Nigeria and Kenya, could better manage supply challenges.^33^,^34^ The statistical significance of increased DMPA-SC consumption during travel restrictions underscores the critical role of self-administration in maintaining contraceptive use. Comparative analyses between periods show that DMPA-SC consumption significantly rose during travel restrictions, as evidenced by data from multiple sources.^30^,^37^ This finding aligns with the observed trends in other self-administered contraceptive methods, where access to traditional healthcare services was limited.^38^,^39^ The studies collectively suggest that enabling self-administration can effectively mitigate disruptions in healthcare access, a valuable insight for future public health strategies. The success of countries that implemented robust community-based distribution and task-shifting policies further supports the importance of these strategies.^34^,^37^ The relationship between movement restrictions and supply availability during the COVID-19 pandemic highlights the importance of maintaining robust supply chains and promoting self-administration options. These strategies can help mitigate the impact of future public health crises on contraceptive access, ensuring sustained and equitable access to reproductive health services.

Supply chain factors such as the percentage of product stock-outs, quantity received, and stock on hand at facilities are critical in understanding the impact of the COVID-19 pandemic on contraceptive access. Studies found that facilities experienced increased stock-outs and supply chain disruptions, affecting contraceptive availability during the pandemic. For instance, a survey of Nigeria’s supply chain indicated significant challenges with maintaining stock levels of DMPA-SC due to disruptions in global supply chains and increased demand.^33^ Similarly, data from WHO indicated that the WHO noted that stock-outs were more frequent during the pandemic, exacerbating the difficulty of maintaining a supply.^37^ The UNFPA reported that countries with robust supply chain management systems, such as Kenya, managed these challenges more effectively by implementing proactive stock monitoring and replenishment strategies.^32^

Additionally, a JSI report on supply chain considerations for DMPA-SC introduction emphasized the risks of mismatched supply and demand, leading to either overstocking or shortages, which can undermine confidence in the supply system.^40^ The increased frequency of stock-outs and supply chain disruptions during the pandemic highlights the vulnerability of contraceptive supply chains to global crises. Studies indicate that facilities with better stock monitoring and management practices were more resilient, as evidenced by the relatively stable contraceptive supplies in countries like Kenya and Nigeria.^32^,^33^ The number of contraceptives received and stock on hand at facilities was crucial to ensure continuous availability. Countries that implemented effective forecasting, inventory management, and emergency stock reserves maintained better supply levels than those that did not.^30^,^37^ The analysis of additional supply chain factors during the COVID-19 pandemic underscores the need for robust supply chain management systems to ensure continuous contraceptive availability. By improving stock monitoring, forecasting, and replenishment strategies, healthcare systems can better withstand global disruptions and maintain consistent access to reproductive health supplies.

This study has several notable strengths that enhance its relevance and robustness. First, it leverages a comprehensive dataset from the national electronic Health Logistics Management Information System (e-HLMIS), covering 26 months of logistics data across 36 states in Nigeria, providing a broad and representative analysis of contraceptive trends during the COVID-19 pandemic. Second, the study employs rigorous statistical methods, including one-way ANOVA and independent samples t-tests, to analyze trends and draw meaningful comparisons between DMPA-SC and other injectable contraceptives, ensuring a data-driven approach to the findings. Third, the study uniquely focuses on the impact of self-administration promotion for DMPA-SC, highlighting a critical innovation in contraceptive delivery that sustained access during a period of global healthcare disruptions. Additionally, by analyzing consumption trends across three distinct phases—pre-restriction, restriction, and post-restriction—it captures the dynamic effects of travel restrictions and healthcare adaptations over time. Finally, the study provides actionable insights for policymakers and healthcare providers, emphasizing the importance of flexible healthcare delivery models and resilient supply chains in maintaining access to essential health services during crises, making its findings highly applicable to similar contexts worldwide.

Some limitations should be considered when interpreting the findings. First, the study relies on secondary data from the national electronic Health Logistics Management Information System (e-HLMIS), which may be subject to reporting inaccuracies, inconsistencies, or incomplete data entries across facilities. Second, while the analysis spans 26 months, it does not account for potential regional variations in healthcare access, policy implementation, or pandemic severity that could have influenced contraceptive uptake. Third, the study focuses on aggregate consumption trends and does not explore individual-level factors, such as user preferences, socioeconomic status, or barriers to access, which may have impacted contraceptive choices. Finally, the study did not account for other possible confounding factors, such as concurrent health campaigns or broader changes in SRH service delivery. These limitations highlight the need for more granular, qualitative, and longitudinal research to comprehensively understand the dynamics of contraceptive use during public health crises.

## Conclusion

This study examined the impact of COVID-19 travel restrictions on the consumption of DMPA-SC and other injectable contraceptives in Nigeria, using data from the national electronic Health Logistics Management Information System (e-HLMIS). The findings demonstrate that DMPA-SC consumption increased significantly during the travel restriction period and continued to rise post-restriction, highlighting the critical role of self-administration in maintaining contraceptive access during health crises. In contrast, other injectable contraceptives, such as DMPA-IM and NET-EN, showed less sustained growth, underscoring the unique adaptability of DMPA-SC to emergency healthcare conditions.

The results further revealed that supply chain factors, particularly the percentage of stock-outs, stock on hand, and quantity received at service delivery points (SDPs), significantly influenced couple-year protection (CYP). Stock-outs were positively associated with CYP, likely reflecting heightened demand during the pandemic, while higher stock levels and timely resupply corresponded to increased contraceptive coverage. However, travel restrictions themselves did not significantly explain CYP variability, suggesting that internal supply chain performance played a more decisive role.

These findings emphasize the importance of flexible healthcare delivery models, such as self-administration, in ensuring the continuity of sexual and reproductive health services during global crises. Strengthening supply chain systems through accurate demand forecasting, real-time inventory monitoring, and proactive replenishment strategies is essential to mitigating the impact of future disruptions on contraceptive access.

For policymakers and healthcare providers, this study underscores the need for sustained investments in self-care interventions and resilient supply chains to enhance the resilience of family planning programs and ensure equitable and uninterrupted access to contraceptives, even in the face of emergencies. Future research should explore individual-level factors influencing contraceptive uptake and assess the long-term implications of self-administration on contraceptive continuation rates and broader health outcomes.

## Operational Definitions

1. *Couple-Year Protection (CYP*): A standard measure of contraceptive coverage representing the estimated years of protection from unintended pregnancy provided by the contraceptives distributed within a specific period. It is calculated based on the quantity of contraceptive products dispensed, using standard conversion factors specific to each method (eg, one unit of DMPA-SC provides three months of protection).
2. *DMPA-SC Consumption*: The total number of units of depot medroxyprogesterone acetate subcutaneous (DMPA-SC) dispensed to clients during a specified period. This metric represents the actual uptake of the contraceptive product.
3. *DMPA-IM Consumption*: The total number of units of depot medroxyprogesterone acetate intramuscular (DMPA-IM) dispensed to clients during a specified period. This serves as a comparative measure for trends in injectable contraceptive uptake.
4. *NET-EN Consumption*: The total number of units of norethisterone enanthate (NET-EN) dispensed to clients during a specified period, included for comparison with other injectable contraceptives.
5. *Percentage of Stock-Outs*: The proportion of service delivery points (SDPs) reporting zero stock of a specific contraceptive (eg, DMPA-SC) at any point during the reporting period, expressed as a percentage of total reporting facilities.
6. *Stock on Hand*: The quantity of contraceptive products (eg, DMPA-SC) available at SDPs at the end of a reporting period. This metric indicates the inventory levels at facilities.
7. *Quantity Received*: The total number of contraceptive units delivered to SDPs during a specific reporting period. This reflects the resupply efforts made to maintain stock availability.
8. *Travel Restriction Period*: The time frame during which government-imposed COVID-19 movement restrictions were in effect, potentially impacting logistics, service delivery, and client access to contraceptives.
9. *Expected Consumption Rate*: The projected number of contraceptive units (eg, DMPA-SC) expected to be consumed during a given period, calculated using a two-period moving average of historical consumption data.
10. *Self-Administration of DMPA-SC*: This delivery model allows clients to inject DMPA-SC independently at home or outside healthcare facilities, reducing reliance on healthcare provider-administered services.
11. *Service Delivery Points (SDPs*): Health facilities or other locations where contraceptives are dispensed to clients. Examples include hospitals, clinics, or community-based distribution points.
12. *Univariate Analysis*: A statistical approach used to examine and summarize data trends for a single variable, such as the mean consumption of DMPA-SC across different time periods.
13. *Health Logistics Management Information System (e-HLMIS*): A digital platform that collects, reports, and analyzes logistics and inventory data related to contraceptive distribution and stock levels across service delivery points.

## Data Availability

The data supporting the results can be accessed through the Nigeria Health Logistics Management Information System (NHLMIS), managed by the Federal Ministry of Health (https://healthlmis.ng/). The FMOH granted the authors’ project access to the platform.

## References

[1] Riley T, Sully E, Ahmed Z, Biddlecom A. Estimates of the potential impact of the COVID-19 pandemic on sexual and reproductive health in low- and middle-income countries. *Int Perspect Sex Reprod Health*. 2020;46:73–76. 32343244 10.1363/46e9020

[2] Purssell E, Balogh R, Lloyd H. Impact of the COVID-19 pandemic on sexual and reproductive health services in low- and middle-income countries: a systematic review. *Glob Public Health*. 2021;16(5):828–839.

[3] Okonofua F, Omo-Aghoja L, Osakue I, Ntoimo L, Balogun J. Covid-19 and Nigeria’s health system: striving for resilience. *Afr J Reprod Health*. 2020;24(2):1–4. doi:10.29063/ajrh2020/v24i4.1 34077065

[4] Hussein J. COVID-19: what implications for sexual and reproductive health and rights globally?. *Sex Reprod Health Matters*. 2020;28(1):1746065. doi:10.1080/26410397.2020.1746065 32191167 PMC7887905

[5] Brolin K, Ekström AM, Nordström A. Maternal and child health in Sierra Leone: the impact of the ebola outbreak. *Glob Health Action*. 2016;9(1):31606.

[6] Jones SA, Gopalakrishnan S, Ameh CA, White S, van den Broek N. Women and babies are dying but not of Ebola: the effect of the Ebola virus epidemic on maternal and neonatal health in Sierra Leone. *PLoS Negl Trop Dis*. 2016;10(6). 10.1136/bmjgh-2016-000065 PMC5321347 28588954

[7] Bietsch K, Williamson J, Reeves M. Family planning during and after the West African Ebola crisis. *Stud Fam Plann*. 2020;51(1):71–86. doi:10.1111/sifp.12110 32180246

[8] Ahmed Z, Sonfield A, Gold RB. The COVID-19 outbreak: potential fallout for sexual and reproductive health and rights. Guttmacher Institute. 2020. Available from: https://www.guttmacher.org/article/2020/03/covid-19-outbreak-potential-fallout-sexual-and-reproductive-health-and-rights. Accessed May 17, 2025.

[9] Dehingia N, Raj A, Silverman J. A conceptual model for the impact of gender inequality on COVID-19 mortality. *Int J Public Health*. 2021;66:1604079.

[10] Carballo M, Heal B, Horbaty G. Impact of the Tsunami on reproductive health. *JAMA*. 2005;293(5):580–581.

[11] Dahlin M. Sexual and reproductive health in emergencies. UNFPA. 2019. Available from: https://www.unfpa.org/sexual-reproductive-health-emergencies. Accessed May 17, 2025.

[12] UNFPA. 450,000 doses of self-injectable contraceptives delivered to support comprehensive access to modern family planning. UNFPA. 2024. Available from: https://kenya.unfpa.org. Accessed May 17, 2025.

[13] Ellington SR, Jamieson DJ, Jones HE. Contraceptive use among women in the United States during disasters. *J Womens Health*. 2013;22(8):703–708.

[14] Hapsari ED, Martiningsih W, Handayani L, Drexler H, Muenster E. Contraceptive use among women in Yogyakarta, Indonesia, after the 2006 earthquake. *BMC Public Health*. 2009;9(1):451. doi:10.1186/1471-2458-9-451 19961624 PMC2797800

[15] Stevenson SM, Wilkins LK, Hainsworth G. Contraceptive supply chain resilience in low- and middle-income countries during the COVID-19 pandemic: lessons learned and the way forward. *Glob Health Sci Pract*. 2021;9(2):272–284.

[16] Adelekan A, Oduro AR, Bolarinwa OA. Impact of COVID-19 on family planning services in Kenya and Nigeria. *Int J Health Plann Manage*. 2021;36(3):732–744.

[17] USAID. Nigeria: family planning supply chain assessment. USAID Global Health Supply Chain Program. 2018. Available from: https://www.ghsupplychain.org/nigeria-family-planning-supply-chain-assessment. Accessed May 17, 2025.

[18] WHO. Maintaining essential health services: operational guidance for the COVID-19 context interim guidance. WHO. 2020. Available from: https://www.who.int/publications/i/item/WHO-2019-nCoV-essential_health_services-2020.1. Accessed May 17, 2025.

[19] National Population Commission (NPC/Nigeria) and ICF International. *Nigeria Demographic and Health Survey*. NPC/Nigeria; 2014.

[20] Darroch JE, Audam S, Biddlecom A. Adding it up: investing in contraception and maternal and newborn health, 2017. Guttmacher Institute. 2017. Available from: https://www.guttmacher.org/report/adding-it-up-investing-in-contraception-maternal-and-newborn-health-2017. Accessed May 17, 2025.

[21] Cover J, Namagembe A, Tumusiime J, Lim J, Drake JK. Acceptability of contraception self-injection with DMPA-SC among adolescents in Uganda. *Glob Health Sci Pract*. 2017;5(4):567–578. 10.1363/43e5117 29771679

[22] Cover J, Ba M, Drake JK, NDiaye MD. Continuation of self-injected versus provider-administered contraception in Senegal: a nonrandomized, prospective cohort study. *Contraception*. 2019;99(2):137–141. doi:10.1016/j.contraception.2018.11.001. 30439358 PMC6367564

[23] Adepoju P. Access to contraceptives in Nigeria during the COVID-19 pandemic. *Lancet Glob Health*. 2021;9(6).

[24] Spieler J. Injectables: safe and effective new delivery options. *Popul Rep*. 2014;27(2):5–12.

[25] FP2020. Nigeria: FP2020 Commitment self-reporting questionnaire. 2019. Available from: https://fp2020.org/resource/nigeria-fp2020-commitment-self-reporting-questionnaire. Accessed May 17, 2025.

[26] Access Collaborative. Nigeria: scaling-up DMPA-SC in public health facilities. Access Collaborative. Available from: https://www.path.org/articles/nigeria-scaling-up-dmpa-sc-public-health-facilities. Accessed May 17, 2025.

[27] Bolarinwa OA, Adelekan A, Oduro AR. The effect of COVID-19 on the use of family planning services in Nigeria. *BMJ Glob Health*. 2022;7(6).

[28] Knowledge Success. Connecting the dots between evidence and experience: impact of COVID-19 on family planning in Africa and Asia. Knowledge Success. Available from: https://knowledgesuccess.org/2022/01/31/introducing-connecting-the-dots-between-covid-19-and-family-planning/. Accessed May 17, 2025.

[29] Rhino. Overview of the Nigerian health logistics management information system (e-HLMIS). Rhino. Available from: https://rhino.africa/e-HLMIS. Accessed May 17, 2025.

[30] PATH. The power to prevent pregnancy in women’s hands: DMPA-SC injectable contraception. PATH. 2023. Available from: https://www.path.org. Accessed May 17, 2025.

[31] UNFPA. Scaling-up community-based counseling and distribution of DMPA-SC in the DRC. Pathfinder International. 2024. Available from: https://www.pathfinder.org. Accessed May 17, 2025.

[32] WHO. Implementation research on DMPA-SC self-injection. WHO. 2023. Available from: https://www.who.int. Accessed May 17, 2025.

[33] BMC Women’s Health. Scale-up of the DMPA-SC in Nigeria: why policy matters. *BMC Women’s Health*. 2023. 10.1186/s12905-022-02109-x PMC9768394 36544189

[34] Pathfinder International. Scaling-up community-based counseling and distribution of DMPA-SC in the DRC. Pathfinder International. 2024. Available from: https://www.pathfinder.org. Accessed May 17, 2025.

[35] Cover J, Lim J, Namagembe A, Tumusiime J, Ba M, Drake JK. Continuation of self-injected versus provider-administered contraception in Senegal and Uganda. *Glob Health Sci Pract*. 2019;7(2):223–233.

[36] Stout A, Wood S, Barigye G, Gregory J. Delivering injectable contraceptives in low-resource settings: a review of the evidence on subcutaneous DMPA. *Glob Health Sci Pract*. 2018;6(3):505–515.

[37] WHO. Implementation research on DMPA-SC self-injection. WHO. 2023. Available from: https://www.who.int. Accessed May 17, 2025.

[38] Aly J, Haeger KO, Christy AY, Johnson AM. Contraception access during the COVID-19 pandemic. *Contracept Reprod Med*. 2020;5(1):1–9. doi:10.1186/s40834-020-00114-9 33042573 PMC7541094

[39] Cartwright AF, Velarde M, Beksinska M, et al. Perspectives on sexual and reproductive health self-care among women, healthcare providers, and other key informants: a mixed-methods study in South Africa and Zambia. *Reprod Health*. 2023;20(1):65. doi:10.1186/s12978-023-01596-x 37118835 PMC10144905

[40] JSI. Supply chain considerations for DMPA-SC introduction: the challenge of matching supply with demand. JSI. 2023. Available from: https://www.jsi.com. Accessed May 17, 2025.

